# Co-Culture of Primary Human Coronary Artery and Internal Thoracic Artery Endothelial Cells Results in Mutually Beneficial Paracrine Interactions

**DOI:** 10.3390/ijms21218032

**Published:** 2020-10-28

**Authors:** Daria Shishkova, Victoria Markova, Maxim Sinitsky, Anna Tsepokina, Alexey Frolov, Nikita Zagorodnikov, Leo Bogdanov, Anton Kutikhin

**Affiliations:** Department of Experimental Medicine, Research Institute for Complex Issues of Cardiovascular Diseases, Kemerovo 650002, Russia; shidk@kemcardio.ru (D.S.); markve@kemcardio.ru (V.M.); sinitsky@kemcardio.ru (M.S.); cepoav@kemcardio.ru (A.T.); frolav@kemcardio.ru (A.F.); zagoni@kemcardio.ru (N.Z.); bogdla@kemcardio.ru (L.B.)

**Keywords:** coronary artery bypass graft surgery, internal thoracic artery, saphenous vein, endothelial cells, paracrine effects, endothelial nitric oxide synthase, endothelial activation, endothelial-to-mesenchymal transition, arterial specification, arterialisation

## Abstract

Although saphenous veins (SVs) are commonly used as conduits for coronary artery bypass grafting (CABG), internal thoracic artery (ITA) grafts have significantly higher long-term patency. As SVs and ITA endothelial cells (ECs) have a considerable level of heterogeneity, we suggested that synergistic paracrine interactions between CA and ITA ECs (HCAECs and HITAECs, respectively) may explain the increased resistance of ITA grafts and adjacent CAs to atherosclerosis and restenosis. In this study, we measured the gene and protein expression of the molecules responsible for endothelial homeostasis, pro-inflammatory response, and endothelial-to-mesenchymal transition in HCAECs co-cultured with either HITAECs or SV ECs (HSaVECs) for an ascending duration. Upon the co-culture, HCAECs and HITAECs showed augmented expression of endothelial nitric oxide synthase (eNOS) and reduced expression of endothelial-to-mesenchymal transition transcription factors Snail and Slug when compared to the HCAEC–HSaVEC model. HCAECs co-cultured with HITAECs demonstrated an upregulation of HES1, a master regulator of arterial specification, of which the expression was also exclusively induced in HSaVECs co-cultured with HCAECs, suggestive of their arterialisation. In addition, co-culture of HCAECs and HITAECs promoted the release of pro-angiogenic molecules. To conclude, co-culture of HCAECs and HITAECs results in reciprocal and beneficial paracrine interactions that might contribute to the better performance of ITA grafts upon CABG.

## 1. Introduction

Coronary artery bypass grafting (CABG) represents the most frequent surgical intervention for the treatment of coronary artery disease [1,2,3,4,5]. Conduits for CABG include autologous veins (e.g., saphenous vein, SV) and arteries (e.g., internal thoracic artery, ITA) [2,3]. Currently, the left ITA is considered the most appropriate vessel to be used first, yet patients often require multiple or composite grafts for complete revascularisation [2,3,6,7]. The selection of the second and further conduits for multiple grafting, as well as those to be included into the composite graft, is a matter of debate [2,3,6,7].

Although arterial grafts generally show better long-term patency (>98%, >95%, and >90% respectively at 1, 5–10, and 15–20 years post-operation) than venous conduits (80%–90%, 50%–75%, and <40% at the same time points) [2,3,6,7,8] because of higher resistance to atherosclerosis, restenosis and thrombosis [2,9,10,11], SV remains the most common second-choice conduit for the CABG, rather than the right ITA [2,3]. The reasons behind this include: (1) less resistance of ITA to injury and vasospasm, which makes the harvesting technique technically challenging; (2) the limited length of the ITA and longer time required for its grafting; (3) a risk of iatrogenic mediastinitis, which is relatively high in patients with comorbid conditions such as obesity or diabetes [12]. However, recent evidence indicates an inverse correlation between the proportion of bilateral ITA grafting across all CABG interventions and long-term mortality [13,14] and supports the use of bilateral ITA for coronary revascularisation in high-risk patients [15] and even in emergency settings [16]. However, the mechanistic explanation of the superior patency of ITAs is generally lacking, thus limiting the justification of this grafting modality.

Endothelial cells (ECs), which secrete a myriad of bioactive factors [17,18,19], show high heterogeneity depending on the type of blood vessel (e.g., arteries and veins) and even on their location in the circulatory system (e.g., CA and ITA) [17,18,20,21,22,23]. Notably, ITA ECs (HITAECs) are characterised by the increased production of endothelial nitric oxide synthase (eNOS) [24], elevated nitric oxide (NO) amounts in response to the vascular endothelial growth factor (VEGF) stimulation [25], and an augmented level of cyclic guanosine monophosphate, which mediates the vasodilating effects of NO [26], in comparison with SV ECs (HSaVECs). Hence, synergistic paracrine interactions between CA ECs (HCAECs) and HITAECs may contribute to the better long-term performance of ITA grafts as compared with SV conduits.

To test the features of HCAEC–HITAEC and HCAEC–HSaVEC interactions in relation to HCAEC homeostasis in an anatomically relevant setting, we co-cultured HCAECs with either HITAECs or HSaVECs for the consecutive time points (6, 24, and 48 h), and then measured the levels of the molecules responsible for endothelial homeostasis and endothelial dysfunction, defined by endothelial activation and endothelial-to-mesenchymal transition (EndoMT) [27,28,29,30,31,32], in all mentioned cell lines. The justification of the selected time points was that confluent EC cultures are metabolically active and undergo serum deprivation in a serum-free medium after 48 h of incubation. Other time points (6 and 24 h) were included in the analysis to better understand the temporal patterns of the molecular response to the co-culture of different EC lines. Among the available molecules, we focused on: (1) eNOS, which represents a key enzyme in the production of nitric oxide (NO), a pivotal vasodilator balancing the vascular tone [33,34]; (2) pro-inflammatory cytokines interleukin-6 (IL-6) and interleukin-8 (IL-8) [35,36,37] and pro-inflammatory cell adhesion molecules vascular cell adhesion molecule 1 (VCAM1), intercellular cell adhesion molecule 1 (ICAM1), and E-selectin, mediating the tethering of monocytes and lymphocytes to the endothelium [38,39]; (3) transcription factors of EndoMT (Snail, Slug, Twist1 and Zeb1) and N-cadherin, a surrogate marker of EndoMT in ECs [40,41].

Co-culture of HCAECs and HITAECs reciprocally promoted the production of eNOS and better maintained endothelial identity, downregulating EndoMT transcription factors and upregulating transcription factors of arterial specification. In accordance with this, co-culture of HCAECs and HSaVECs induced arterial differentiation of the latter. Further, co-culture of HCAECs and HITAECs resulted in the augmented release of pro-angiogenic molecules into the microenvironment, which might contribute to vascular regeneration. We suggest that the paracrine interactions between HCAECs and ECs of its bypass graft may affect vascular homeostasis after CABG and might partially explain the better long-term performance of ITA grafts.

## 2. Results

To investigate whether HITAECs, but not HSaVECs, induce advantageous paracrine effects for HCAECs, and whether each of these intercellular interactions has a distinct pattern of detrimental and beneficial effects, we applied a co-culture model where HCAECs were settled at the bottom of a cell culture dish while conduit ECs (HITAECs or HSaVECs) were located at the surface of a 10-μm-thick translucent polycarbonate membrane insert. The pore diameter of this insert (0.4 μm) permitted the communication of co-cultured cells by means of soluble factors and extracellular vesicles while excluding cell migration. Therefore, cell lines were reliably separated, yet still constantly affecting the behaviour of each other through paracrine signals. Sequential time points of the supernatant collection with subsequent RNA/protein extraction after the cell lysis allowed for the assessment of the temporal expression patterns in HCAECs from both of the co-culture models and also in HITAECs/HSaVECs. Monocultures of HCAECs, HITAECs, and HSaVECs were used as a kind of control.

**Figure 1:**
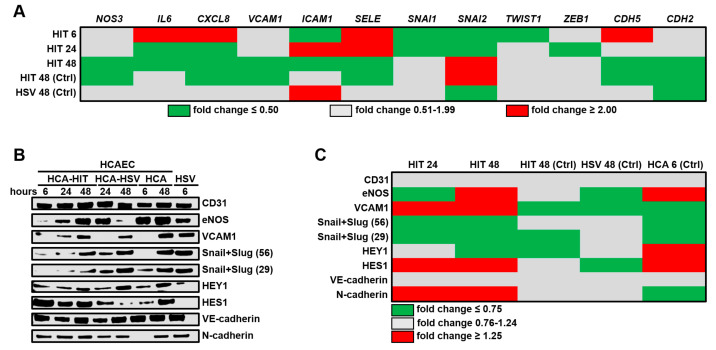
Profiling of key endothelial molecules in human coronary artery endothelial cells (HCAECs) co-cultured with either human internal thoracic artery endothelial cells (HITAECs) (HIT) or human saphenous vein endothelial cells (HSaVECs) (HSVs) for 6, 24, or 48 h. (**A**) RT-qPCR profiling, HIT signifies the ratio of transcript levels (measured as ΔCt) in HCAECs co-cultured with HITAECs to those in HCAECs co-cultured with HSaVECs. HIT (Ctrl) and HSV (Ctrl) represent the ratios of transcript levels (measured as ΔCt) in HCAECs co-cultured with HITAECs or HSaVECs to those in HCAEC monoculture. Results are represented as the heat map; green, gray, and red colours indicate fold change ≤0.50, 0.51–1.99, and ≥2.00, respectively; (**B**) Western blotting measurements. HCA-HIT represents HCAECs co-cultured with HITAECs, HCA-HSV signifies HCAECs co-cultured with HSaVECs. HCA and HSV signify HCAEC and HSaVEC monocultures, respectively; (**C**) Semi-quantitative analysis of Western blotting results by densitometry. HIT represents the ratio of band density in HCAECs co-cultured with HITAECs to that in HCAECs co-cultured with HSaVECs. HIT (Ctrl) and HSV (Ctrl) abbreviate the ratios of band density in HCAECs co-cultured with HITAECs or HSaVECs to that in HCAEC monoculture. HCA (Ctrl) abbreviates the ratio of band density in HCAEC monoculture to that in HSaVEC monoculture. Snail + Slug are shown at both 56 and 29 kDa values. Results are represented as green, gray, and red colours on the the heat map, indicating fold changes of ≤0.75, 0.76–1.24, and ≥1.25, respectively.

We first investigated whether co-culturing with HITAECs or HSaVECs prevents or contributes to the development of endothelial dysfunction in HCAECs by measuring the relative levels of eNOS (*NOS3* gene), pro-inflammatory cytokines IL-6 (*IL6* gene), and IL-8 (*CXCL8* gene), molecules which are responsible for the monocyte/lymphocyte attachment to ECs (VCAM1/*VCAM1*/, ICAM1/*ICAM1*, and E-selectin (SELE/*SELE*)), EndoMT transcription factors (Snail/*SNAI1*, Slug/*SNAI2*, Twist1/*TWIST1*, and Zeb1/*ZEB1*), EndoMT markers (endothelial marker VE-cadherin/*CDH5* and mesenchymal marker N-cadherin/*CDH2*), and arterial specification transcription factors (HEY1 and HES1). Selective gene expression profiling by means of reverse transcription-quantitative polymerase chain reaction (RT-qPCR) found that pro-inflammatory and EndoMT transcripts were underrepresented in HCAECs upon co-culturing with HITAECs at different time points as compared to the HCAEC–HSaVEC co-culture model (Figure 1A). For instance, *IL6* and *CXCL8* genes were downregulated in HCAECs at both 24 and 48 h of co-culture with HITAECs, whereas *VCAM1*, *ICAM1*, *SELE*, *SNAI1*, and *SNAI2* genes were differentially expressed at these time points (Figure 1A). Downregulation of *SNAI1* and *SNAI2* genes was noted at 6 and 24 h, followed by a decrease in the expression of *VCAM1*, *ICAM1*, and *SELE* genes, which encode the respective cell adhesion molecules (Figure 1A). No beneficial or detrimental pattern was identified when comparing the abundance of abovementioned transcripts in HCAECs from both co-culture models and HCAECs cultured separately (Figure 1A).

Measurement of the corresponding proteins by Western blotting (Figure 1B) found an increase in the eNOS level in HCAECs co-cultured with HITAECs when compared to the HCAEC–HSaVEC model upon 48 h of co-culture (Figure 1B,C). Unexpectedly, the level of VCAM1 was also augmented, yet still being lower in both co-cultures than in the monocultures (Figure 1B,C). The level of Snail and Slug, however, was steadily reduced in HCAECs co-cultured with HITAECs, corroborating the results of the gene expression analysis (Figure 1B,C). Together with the upregulated transcription factor of arterial specification HES1, this might indicate that HITAECs supply HCAECs with supportive arterial differentiation cues, whereas HSaVECs deregulate the maintenance of arterial differentiation in HCAECs through paracrine signaling. Notably, both CD31 and vascular endothelial (VE) cadherin expression remained unaffected, suggesting that the alterations of endothelial identity occur far beyond the investigated time points.

**Figure 2:**
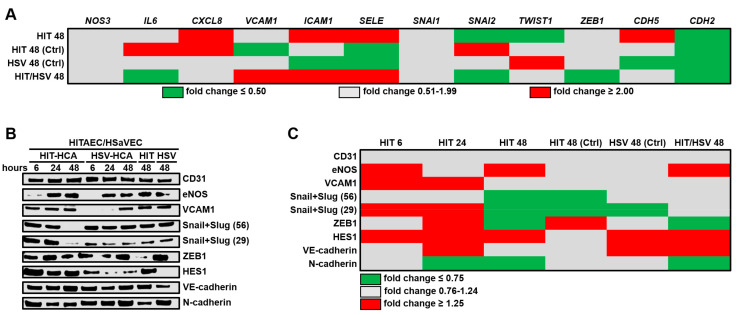
Profiling of key endothelial molecules in HITAECs (HIT) and HSaVECs (HSV) co-cultured with HCAECs for 6, 24, or 48 h. (**A**) RT-qPCR profiling, HIT signifies the ratio of transcript levels (measured as ΔCt) in HITAECs to those in HSaVECs co-cultured with HCAECs. HIT (Ctrl) and HSV (Ctrl) represent the ratios of transcript levels (measured as ΔCt) in HITAECs and HSaVECs co-cultured with HCAECs to those in the respective monocultures. HIT/HSV signifies the ratio of transcript levels (measured as ΔCt) in HITAEC to those in HSaVEC monocultures. Results are represented as the heat map; green, gray, and red colours indicate fold changes of ≤0.50, 0.51–1.99, and ≥2.00, respectively; (**B**) Western blotting measurements. HIT-HCA signifies HITAECs co-cultured with HCAECs, HSV-HCA abbreviates HSaVECs co-cultured with HCAECs. HIT and HSV represent HITAEC and HSaVEC monocultures, respectively. (**C**) Semi-quantitative analysis of Western blotting results by densitometry. HIT signifies the ratio of band density in HITAECs to that in HSaVECs co-cultured with HCAECs. HIT (Ctrl) and HSV (Ctrl) signify the ratios of band density in HITAECs and HSaVECs co-cultured with HCAECs to those in the respective monocultures. HIT/HSV signifies the ratio of band density in HITAEC monoculture to that in HSaVEC monoculture. Snail + Slug are shown at both 56 and 29 kDa values. Results are represented as green, gray, and red colours on the heat map, indicating fold changes of ≤0.75, 0.76–1.24, and ≥1.25, respectively.

We next assessed whether HCAECs differentially affect HITAECs and HSaVECs during the co-culture. Expression of pro-inflammatory genes (*CXCL8*, *ICAM1*, and *SELE*) was higher in HITAECs than in HSaVECs, whereas EndoMT transcription factors *SNAI2* and *TWIST1* were downregulated, along with the *CDH2* gene encoding N-cadherin (Figure 2A). In contrast, the *CDH5* gene encoding VE-cadherin was overexpressed in HITAECs (Figure 2A). Having compared HITAECs and HSaVECs co-cultured with HCAECs with the respective monocultures, we found that co-culture with HCAECs repressed the expression of the *VCAM1*, *ICAM1*, and *SELE* genes in HITAECs and HSaVECs, although simultaneously promoting *IL6* and *CXCL8* gene expression in HITAECs (Figure 2A). Interestingly, the monoculture of HITAECs showed a higher expression of cell adhesion molecule genes (*VCAM1*, *ICAM1*, and *SELE*) and a lower expression of EndoMT markers (*SNAI2*, *ZEB1*, and *CDH2* genes) than HSaVECs (Figure 2A).

Similar to HCAECs, HITAECs from the arterial endothelial co-culture model were characterised by an upregulation of eNOS and VCAM1 proteins (Figure 2B,C). Strikingly, co-culture with HCAECs abrogated the expression of Snail and Slug in HITAECs and endowed HSaVECs with HES1 expression, suggestive of HSaVEC arterialisation during the co-culture with HCAECs (Figure 2B,C). In addition, the expression of VE-cadherin in HSaVECs also increased upon the co-culture with HCAECs (Figure 2B,C). In accord with earlier reports [24], HITAECs demonstrated higher eNOS expression than HSaVECs and also had a higher VE-cadherin/N-cadherin ratio (Figure 2B,C).

Taken together, patterns of molecular response in both co-culture models significantly changed over time but indicated mutual benefits of the paracrine interactions between HCAECs and HITAECs. Notable discrepancies between the expression of the measured molecules at the gene and protein level suggested significant involvement of post-transcriptional regulation.

**Figure 3:**
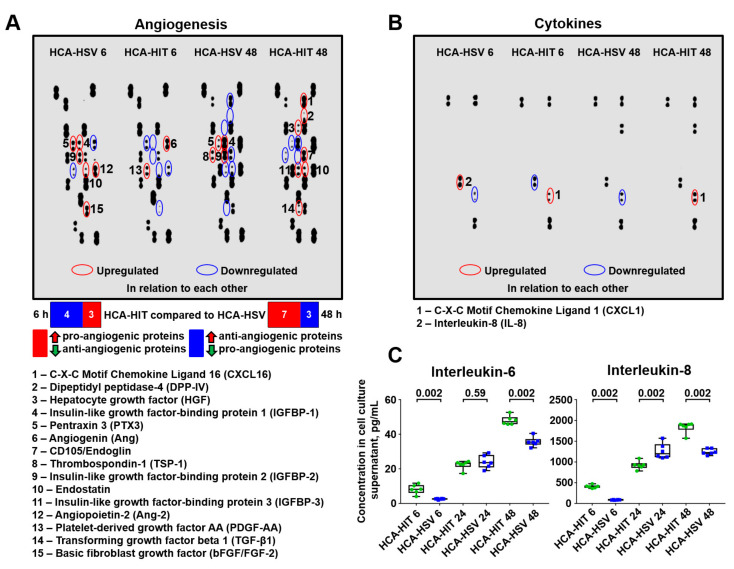
Secretome profiling in the supernatants from HCAEC–HITAEC and HCAEC–HSaVEC co-culture models collected at 6- and 48-h time points. (**A**) Measurement of 55 human angiogenesis-related proteins. (**B**) Measurement of 36 cytokines, chemokines, and acute phase proteins. Those upregulated or downregulated in the HCAEC–HITAEC (HCA-HIT) co-culture model in comparison with the HCAEC–HSaVEC (HCA-HSV) model are circled in red and blue, respectively. (**C**) High-sensitivity enzyme-linked immunosorbent assay measurements of interleukin-6 and interleukin-8. For this experiment, we also included a 24-h time point. Each dot represents one aliquot from the co-culture model. Whiskers indicate range, boxes’ bounds indicate 25th–75th percentiles, center lines indicate median. *P* values provided above boxes, Mann–Whitney U-test.

Finally, we evaluated the changes in the EC secretome in different co-culture setups. Pro-angiogenic proteins tended to be upregulated at 48 h in the HCAEC/HITAEC supernatant, in comparison with HCAEC/HSaVEC supernatant, although this was not the case at the 6-h time point (Figure 3A). Notably, the level of the major pro-inflammatory cytokine interleukin-8 in the supernatant was comparable across the co-culture models, although being slightly higher in the HCAEC/HSaVEC supernatant at the 6-h time point (Figure 3B). However, a more sensitive enzyme-linked immunosorbent assay identified increased IL-6 and IL-8 content in the supernatant from the HCAEC–HITAEC co-culture model (Figure 3C). Collectively, our results support the hypothesis regarding the beneficial, although time-dependent, effects of HCAEC and HITAEC co-culturing. In other words, HCAECs and HITAECs reciprocally induce a favourable expression pattern.

## 3. Discussion

Despite numerous clinical studies [7,8,9,14,15,16] having been performed and many narrative [2,3,12] and systematic reviews [6,13] having been published, there is no clear consensus regarding the choice of the second-best graft for CABG surgery. Among all vessels, right ITAs and SVs are by far the most frequently used in this regard. Although the majority of CABG interventions rely on the SV because of its relatively easy harvest, resistance to manipulation and vasospasm, shorter operative time, and lower risk of iatrogenic complications, which collectively lead to better short-term results, an increasing number of reports indicates the advantages of bilateral ITA grafting, which is associated with significantly higher long-term patency [13,14,15,16]. The advantages of ITA include its superior mechanical properties, as it shares most of the histoarchitecture features of CAs, including multiple layers of vascular smooth muscle cells enclosed by two elastic membranes [11]. However, the diameter of the SV (4–5 mm) [42,43,44] is generally similar to the left main CA (4–5 mm) or proximal left anterior descending (LAD) CA (4 mm) [45,46] in contrast to ITA (2–3 mm) [44,47,48,49], although the diameter of the latter is identical to the distal LAD artery (2 mm) [45]. The diameter of the CA, however, may vary from 2 to 5 mm at all segments [46] and therefore individual anatomical features of both the CA and candidate conduits should be taken into account to select an optimal graft for CABG surgery. Nonetheless, elevated release of NO by HITAECs and the correspondingly increased vasoreactivity of the ITA [24,25,26] provide for its higher vasodilation in response to the biochemical cues and blood pressure alterations [25,50,51,52].

Although the short-term benefits of the SV conduits are clearly associated with their convenience for surgical handling, the long-term advantages of ITA grafts are determined by their physiological features, which might include better integration of the HITAEC and HCAEC endothelial layers. In other words, the interactions between HCAECs and HITAECs may sustain coronary homeostasis, whereas those between HCAECs and HSaVECs may fail to endow the corresponding vessels with resistance to restenosis and atherosclerosis. Another scenario implies differential patterns of beneficial and deleterious effects exhibited by each of the EC combinations (HCAEC–HITAEC and HCAEC–HSaVEC). In this study, we attempted to simulate the interactions between HCAECs and conduit ECs (HITAECs and HSaVECs), employing a co-culture model and measuring the expression of key endothelial genes and proteins, as well as secreted factors.

Co-culture of HCAECs and HITAECs led to the increased production of eNOS, reduced expression of EndoMT transcription factors Snail and Slug, and upregulation of arterial specification transcription factor HES1 in HCAECs in pairwise comparison with the HCAEC–HSaVEC co-culture model. Importantly, co-culture with HCAECs augmented eNOS expression and concurrently abrogated the synthesis of Snail and Slug in HITAECs, while bestowing HSaVECs with an arterial signature protein, HES1. Taken together, these observations testify to the synergistic and mutually favourable interactions between HCAECs and their conduit ECs, in particular HITAECs. It also suggests an additional mechanistic explanation for the arterialisation of SV conduits in the heterotopic position upon CABG in addition to pulsatile flow, increased blood pressure, higher shear stress, and oxygen-rich blood [12]. 

Secretion of the angiogenic factors was differentially regulated over time in both co-culture models. For instance, at the 6-h time point, the supernatant collected from a HCAEC and HITAEC co-culture was not characterised by a clear pro- or anti-angiogenic profile, having reduced levels of pro-angiogenic proteins pentraxin 3 [53,54], insulin-like growth factor binding protein-2 [55,56], angiopoietin-2 [57,58], and basic fibroblast growth factor [59,60], as well as lower level of anti-angiogenic protein endostatin [61,62] and higher levels of pro-angiogenic molecules angiogenin [63,64] and platelet-derived growth factor AA [65,66] in comparison with a supernatant from the HCAEC and HSaVEC co-culture. However, at 48 h of HCAEC and HITAEC co-culture, we detected an increased release of multiple pro-angiogenic proteins such as CXCL16 [67,68], dipeptidyl peptidase 4 [69,70], hepatocyte growth factor [71,72], CD105/endoglin [73,74], insulin-like growth factor binding protein-3 [75,76], and transforming growth factor-beta 1 [77,78], as well as reduced secretion of anti-angiogenic protein thrombospondin 1 [79,80] compared to the HCAEC–HSaVEC supernatant, which might contribute to vascular regeneration after the intervention. However, the secretion of anti-angiogenic protein endostatin [61,62] was upregulated, whereas pro-angiogenic proteins pentraxin 3 [53,54] and insulin-like growth factor binding protein-2 [55,56] were downregulated in the HCAEC–HITAEC co-culture model at this time point, also pointing to the mechanisms balancing this pro-angiogenic shift. 

To date, the exact mechanisms of these paracrine effects are obscure and remain to be investigated in detail. Nevertheless, eNOS acts as a potent stimulator of angiogenesis in animal models of experimental ischaemia [81,82,83,84], being reciprocally enhanced by VEGF [85,86,87]. Another critical angiogenic regulator is the Notch pathway [88,89] which also contributes to the production of eNOS [90] and interacts with VEGF in EC sprouting [91,92,93]. We suggest that the relative increase in pro-angiogenic molecules at 48 h in the HCAEC–HITAEC co-culture setup might be related to the upregulation of eNOS and HES1, a master regulator of the Notch pathway (as compared to the HCAEC–HSaVEC model). In addition to the pro-angiogenic switch, an increased expression of eNOS in a HCAEC–HITAEC co-culture setup might explain both the reduction in EndoMT transcription factors and the paradoxical increase in IL-6 and IL-8 secretion, as the loss of eNOS promotes [94] and accompanies [95,96] EndoMT, whereas its upregulation potentiates inflammation [97,98].

Communication of HCAECs and HITAECs (or HSaVECs) upon CABG surgery may be carried out by extracellular vesicles (microvesicles, exosomes, and exomeres), which transfer cargo between the cells through the circulatory system [99,100,101,102,103], or by secreted molecules such as IL-8, a major pro-inflammatory cytokine [104]. As such, endothelial extracellular vesicles transport angiogenic mRNA (VEGF, basic fibroblast growth factor, stromal cell-derived factor/CXCL12), supporting endothelial homeostasis [105,106,107,108]. In endothelial dysfunction, the profile of extracellular vesicles shifts from anti-apoptotic, anti-inflammatory and pro-angiogenic to pro-apoptotic, pro-inflammatory, and anti-angiogenic, promoting the development of the pathological microenvironment [109,110]. Mechanical and biochemical alterations induced by the artificial anastomosis of the CA with SVs may negatively affect the endothelium, probably triggering adverse changes in the profile of extracellular vesicles secreted by HCAECs and conduit ECs. In contrast, total arterial revascularisation may support endothelial homeostasis and preserve physiological extracellular vesicle profile. Further studies are required to explore whether the extracellular vesicles are behind the beneficial interactions between HCAECs and HITAECs revealed in this investigation. 

The drawbacks of our model include the absence of other cell populations which could contribute to paracrine signaling (e.g., vascular smooth muscle cells) and the limited duration of the time-lapse analysis (from 6 to 48 h), which could affect the results due to the specific temporal expression patterns we have shown. The use of blood vessel explants could be an appropriate solution, as they are widely established in atherosclerosis research [111,112,113] and CABG conduits are also investigated ex vivo [114,115]. However, this is barely possible for the CA. Regarding the time points, confluent EC cultures undergo active metabolism and therefore the medium should be collected within the 48 h maximum to ensure the proper maintenance of endothelial homeostasis in serum-free conditions. Hence, we believe the co-culture model is suitable for the in vitro analysis of possible interactions between HCAECs and conduit ECs. Nonetheless, co-culture of arterial ECs with the respective (for instance, coronary and internal thoracic artery) vascular smooth muscle cells and subsequent profiling of their transcriptome, proteome, and secretome would be useful to interrogate how the interactions of ECs with other vascular populations may modulate blood vessel homeostasis upon CABG. Another intriguing issue is that the results of RT-qPCR and Western blotting profiling considerably differed across the time points. However, this may be explained by the involvement of post-transcriptional regulation (e.g., by miRNA or translation initiation factors) and has been observed previously during the comparison of endothelial differentiation signatures defined by RNA-seq and different Western blotting modalities [116].

In conclusion, we suggest that the paracrine interactions between the CA and its bypass graft (in particular ITA) ECs after CABG might be reciprocal and beneficial, probably contributing to the better long-term patency of the ITA grafts following CABG. Further studies in this direction would identify the patterns and mechanisms of this synergy.

## 4. Materials and Methods 

### 4.1. Co-Culture Model

Confluent (85%–90%) HCAECs (300K-05a, Cell Applications, San Diego, CA, USA) were co-cultured with either HITAECs (308K-05a, Cell Applications, San Diego, CA, USA) or HSaVECs (C-12231, PromoCell, Heidelberg, Germany) in co-culture chambers (CLS3419-12EA, Sigma-Aldrich, St. Louis, MO, USA) filled with MesoEndo Basal Medium (210–500, Cell Applications, San Diego, CA, USA). As a kind of control, we used HCAEC, HITAEC, and HSaVEC monocultures grown separately in the same type of the dish. All cell culture procedures were performed strictly under sterile conditions. For the whole time of the experiment, cell cultures were incubated at physiological temperature (37 °C), 95% air:5% CO_2_ atmosphere, and high humidity. Upon 6, 24, or 48 h of co-culture, we collected the conditioned medium to measure the levels of cytokines and angiogenic molecules.

After the supernatant collection, cells were washed with ice-cold phosphate-buffered saline (PBS) and lysed with TRIzol Reagent (15596018, Thermo Fisher Scientific, Waltham, MA, USA) for RNA extraction or with a radioimmunoprecipitation (RIPA) assay buffer (89901, Thermo Fisher Scientific, Waltham, MA, USA) supplied with Halt protease and phosphatase inhibitor cocktail (78444, Thermo Fisher Scientific, Waltham, MA, USA) for the total protein extraction, according to the manufacturer’s protocols. Quantification and quality control of the isolated RNA was performed employing a Qubit 4 fluorometer (Q33238, Thermo Fisher Scientific, Waltham, MA, USA), Qubit RNA BR assay kit (Q10210, Thermo Fisher Scientific, Waltham, MA, USA), Qubit RNA IQ assay kit (Q33222, Thermo Fisher Scientific, Waltham, MA, USA), Qubit RNA IQ standards for calibration (Q33235, Thermo Fisher Scientific, Waltham, MA, USA), and Qubit assay tubes (Q32856, Thermo Fisher Scientific, Waltham, MA, USA), according to the manufacturer’s protocols. Quantification of total protein was conducted using a BCA Protein Assay Kit (23227, Thermo Fisher Scientific, Waltham, MA, USA) and a Multiskan Sky microplate spectrophotometer (51119700DP, Thermo Fisher Scientific, Waltham, MA, USA) in accordance with the manufacturer’s protocols.

### 4.2. RT-qPCR

Reverse transcription was carried out utilising a High Capacity cDNA Reverse Transcription Kit (4368814, Thermo Fisher Scientific, Waltham, MA, USA). Gene expression was measured by RT-qPCR using customised primers (Table 1) (500 nmol/L each, Evrogen, Moscow, Russian Federation, Table I), cDNA (20 ng), and the PowerUp SYBR Green Master Mix (A25778, Thermo Fisher Scientific, Waltham, MA, USA), according to the manufacturer’s protocols for Tm ≥60 °C (fast cycling mode). Technical replicates (*n* = 3 per each sample) were performed in all RT-qPCR experiments. The reaction was considered successful if its efficiency was 90%–105% and R^2^ was ≥0.98. Quantification of the mRNA levels (*NOS3*, *IL6*, *CXCL8*, *VCAM1*, *ICAM1*, *SELE*, *SNAI1*, *SNAI2*, *TWIST1*, *ZEB1*, *CDH5*, *CDH2*) was performed using the 2^−ΔΔCt^ method. Relative transcript levels were expressed as a value relative to the endothelial housekeeping gene *PECAM1* and to the reference group (2^−ΔΔCt^). These values were finally represented as a heat map (green, gray, and red colours reflected fold changes of ≤0.50, 0.51–1.99, and ≥2.00, respectively).

### 4.3. Western Blotting

Equal amounts of protein (10 μg per sample) were mixed with NuPAGE lithium dodecyl sulfate sample buffer (NP0007, Thermo Fisher Scientific, Waltham, MA, USA) at a 4:1 ratio and NuPAGE sample reducing agent ((NP0009, Thermo Fisher Scientific, Waltham, MA, USA) at a 10:1 ratio, denatured at 99 °C for 5 min, and then loaded on 1.5-mm NuPAGE 4%–12% Bis-Tris protein gel ((NP0335BOX, Thermo Fisher Scientific, Waltham, MA, USA). The 1:1 mixture of Novex Sharp pre-stained protein standard (LC5800, Thermo Fisher Scientific, Waltham, MA, USA) and MagicMark XP Western protein standard (LC5602, Thermo Fisher Scientific, Waltham, MA, USA) was loaded as a molecular weight marker. Proteins were separated using sodium dodecyl sulphate-polyacrylamide gel electrophoresis (SDS-PAGE) at 150 V for 2 h using NuPAGE 2-(N-morpholino)ethanesulfonic acid SDS running buffer (NP0002, Thermo Fisher Scientific, Waltham, MA, USA), NuPAGE Antioxidant (NP0005, Thermo Fisher Scientific, Waltham, MA, USA), and an XCell SureLock Mini-Cell vertical mini-protein gel electrophoresis system (EI0001, Thermo Fisher Scientific, Waltham, MA, USA). Protein transfer was performed using polyvinylidene difluoride (PVDF) transfer stacks (IB24001, Invitrogen) and an iBlot 2 Gel Transfer Device (Invitrogen), according to the manufacturer’s protocols, using a standard transfer mode for 30–150 kDa proteins (P0–20 V for 1 min, 23 V for 4 min, and 25 V for 2 min). PVDF membranes were then incubated in iBind Flex Solution (SLF2020, Solution Kit Thermo Fisher Scientific, Waltham, MA, USA) for 1 h to prevent non-specific binding.

Blots were probed with rabbit antibodies to VCAM1 (ab134047, 1:1000, Abcam, Cambridge, UK), Snail and Slug (ab180714, 1:500, Abcam, Cambridge, UK), HEY1 (ab154077, 1:200, Abcam, Cambridge, UK), HES1 (ab108937, 1:200, Abcam, Cambridge, UK), ZEB1 (ab203829, 1:200, Abcam, Cambridge, UK) and VE-cadherin (361900, 1:100, Thermo Fisher Scientific, Waltham, MA, USA), or mouse antibodies to CD31 (loading control, ab9498, 1:1000, Abcam, Cambridge, UK), eNOS (ab76198, 1:500, Abcam, Cambridge, UK) and N-cadherin (MA515633, 1:500, Thermo Fisher Scientific, Waltham, MA, USA). Horseradish peroxidase-conjugated goat anti-rabbit (7074, Cell Signaling Technology, Danvers, MA, USA) or goat anti-mouse (AP130P, Sigma-Aldrich, St. Louis, MO, USA) secondary antibodies were used at 1:200 and 1:1000 dilutions, respectively.

Incubation with the antibodies was performed using iBind Flex Solution Kit (SLF2020, Thermo Fisher Scientific, Waltham, MA, USA), iBind Flex Cards (SLF2010, Thermo Fisher Scientific, Waltham, MA, USA), and an iBind Flex Western Device (SLF2000, Thermo Fisher Scientific, Waltham, MA, USA) for 3 h, according to the manufacturer’s protocols. Chemiluminescent detection was performed using SuperSignal West Pico PLUS chemiluminescent substrate (34580, Thermo Fisher Scientific, Waltham, MA, USA) and a C-DiGit blot scanner (LI-COR Biosciences, Linkoln, NE, USA) in a high-sensitivity mode (12-min scanning). Densitometry was performed using ImageJ software (National Institutes of Health, Bethesda, MD, USA) using the standard algorithm (consecutive selection and plotting of the lanes with the measurement of the peak area and subsequent adjustment for the loading control (CD31) and reference group). The adjusted densitometry values were finally represented as a heat map (green, gray, and red colours reflected fold changes of ≤0.75, 0.76–1.24, and ≥1.25, respectively).

### 4.4. Secretome Profiling

Conditioned medium from both co-culture models collected at 6- and 48-h time points was profiled for angiogenic factors and cytokines, employing the respective dot blotting kits (ARY007 and ARY005B, R&D Systems, Minneapolis, MN, USA) according to the manufacturer’s protocols. Chemiluminescent detection was performed using a C-DiGit blot scanner (LI-COR Biosciences, Linkoln, NE, USA) in a high-sensitivity mode (12-min scanning). Additionally, the concentrations of IL-6 and IL-8 in the cell culture supernatant were analysed utilising respective enzyme-linked immunosorbent assay kits (ab178013 and ab46032, Abcam, Cambridge, UK), according to the manufacturer’s protocols. The colorimetric detection was carried out utilising a Multiskan Sky microplate spectrophotometer (51119700DP, Thermo Fisher Scientific, Waltham, MA, USA) at a 450-nm wavelength.

### 4.5. Statistical Analysis

Statistical analysis was performed in GraphPad Prism 7 (GraphPad Software, San Diego, CA, USA). For descriptive statistics, data were represented by the median, 25th, and 75th percentiles, and range. Groups were compared by means of the Mann–Whitney U-test. *P* values ≤ 0.05 were regarded as statistically significant.

## 5. Conclusions

Paracrine interactions between the CA and its bypass graft (in particular ITA) ECs after CABG might be reciprocal and beneficial, probably contributing to the better long-term patency of the ITA grafts following CABG.

## Figures and Tables

**Table 1:** Sequences of customised primers for RT-qPCR.

Gene	Forward Primer Sequence	Reverse Primer Sequence
*NOS3*	5′-GTGATGGCGAAGCGAGTGAAG-3′	5′-CCGAGCCCGAACACACAGAAC-3′
*IL6*	5′-GGCACTGGCAGAAAACAACC-3′	5′-GCAAGTCTCCTCATTGAATCC-3′
*CXCL8*	5′-CAGAGACAGCAGAGCACAC-3′	5′-AGTTCTTTAGCACTCCTTGGC-3′
*VCAM1*	5′-CGTCTTGGTCAGCCCTTCCT-3′	5′-ACATTCATATACTCCCGCATCCTTC-3′
*ICAM1*	5′-TTGGGCATAGAGACCCCGTT-3′	5′-GCACATTGCTCAGTTCATACACC-3′
*SELE*	5′-GCACAGCCTTGTCCAACC-3′	5′-ACCTCACCAAACCCTTCG-3′
*NAI1*	5′-CAGACCCACTCAGATGTCAAGAA-3′	5′-GGGCAGGTATGGAGAGGAAGA-3′
*SNAI2*	5′-ACTCCGAAGCCAAATGACAA-3′	5′-CTCTCTCTGTGGGTGTGTGT-3′
*TWIST1*	5′-GTCCGCAGTCTTACGAGGAG-3′	5′-GCTTGAGGGTCTGAATCTTGCT-3′
*ZEB1*	5′-GATGATGAATGCGAGTCAGATGC-3′	5′-ACAGCAGTGTCTTGTTGTTGT-3′
*CDH5*	5′-AAGCGTGAGTCGCAAGAATG-3′	5′-TCTCCAGGTTTTCGCCAGTG-3′
*CDH2*	5′-GCTTCTGGTGAAATCGCATTA-3′	5′-AGTCTCTCTTCTGCCTTTGTAG-3′
*PECAM1*	5′-TGGCGCATGCCTGTAGTA-3′	5′-TCCGTTTCCTGGGTTCAA-3′

## Abbreviations

CABG	Coronary artery bypass grafting
SV	Saphenous vein
ITA	Internal thoracic artery
ECs	Endothelial cells
HITAEC	Human internal thoracic artery endothelial cells
eNOS	Endothelial nitric oxide synthase
VEGF	Vascular endothelial growth factor
HSaVEC	Human saphenous vein endothelial cells
HCAEC	Human coronary artery endothelial cells
EndoMT	Endothelial-to-mesenchymal transition
RNA	Ribonucleic acid
IL	Interleukin
CXCL	Chemokine (C-X-C motif) ligand
VCAM	Vascular cell adhesion molecule
ICAM	Intercellular cell adhesion molecule
SELE	E-selectin
SNAI	Snail family transcriptional repressor
TWIST	Twist family basic helix-loop-helix transcription factor
ZEB	Zinc finger E-box binding homeobox
CDH	Cadherin
VE-cadherin	Vascular endothelial cadherin
N-cadherin	Neural cadherin
HEY	Hairy enhancer of split-related family basic helix-loop-helix transcription factor with YRPW motif
HES	Hairy enhancer of split family helix-loop-helix transcription factor
RT-qPCR	Reverse transcription-quantitative polymerase chain reaction
CD	Cluster of differentiation
LAD	Left anterior descending coronary artery
PBS	Phosphate-buffered saline
RIPA	Radioimmunoprecipitation assay
cDNA	Complementary DNA
PECAM	Platelet endothelial cell adhesion molecule
